# Using the Flexor Reflex in a Chronic Stroke Patient for Gait Improvement: A Case Report

**DOI:** 10.3389/fneur.2021.691214

**Published:** 2021-06-18

**Authors:** Christian Salzmann, Aida Sehle, Joachim Liepert

**Affiliations:** ^1^Kliniken Schmieder, Allensbach, Germany; ^2^Lurija Institute, Allensbach, Germany

**Keywords:** stroke, flexor reflex, gait, training, kinematic analysis

## Abstract

The flexor reflex or withdrawal reflex can be elicited by electrical stimulation of the sole of the foot, which serves as a reflex to protect the stimulated limb against tissue damage and consists of flexion movements in the hip, knee, and ankle joint. Triggering this reflex might improve walking abilities in hemiparetic patients. We report the first case of a chronic stroke patient with the most severe impairment of walking. She was examined with and without flexor reflex activation by the Incedo® system. Tests included a 10-m walk and a 2-min walk at baseline, after 3 weeks of training with the Incedo® system and after a follow-up 3 weeks later. Moreover, a kinematic gait analysis was done before and after the training period. At baseline, activation of the flexor reflex induced an improved gait velocity. After the training period, the patient walked twice as fast compared with baseline. Her gait velocity without Incedo® was faster than the gait velocity with Incedo® at baseline. Examination at follow-up indicated that the improvements remained almost unchanged. The kinematic analysis showed an improved stride length and gait velocity during flexor reflex activation. Initially, the foot was elevated higher above the ground during flexor reflex activation. In conclusion, this first case report of a chronic stroke patient demonstrates that flexor reflex activation is feasible and improves gait parameters despite severe impairment of walking abilities.

## Introduction

The flexor reflex (FR) is a polysynaptic and multisegmental spinal response that produces a withdrawal of the stimulated limb in order to protect against tissue damage from contact with noxious stimuli (1–3). It can be elicited by electrical stimulation of the sole of the foot and consists of flexion movements in the hip, knee, and ankle joint. Application of the FR in hemiparetic patients after subacute stroke has been shown to improve walking velocity (4).

This report deals with a chronic stroke patient most severely affected in her ability to walk. We explored if the use of the FR changed gait velocity, walking distance, and kinematic parameters even during the first application and if eliciting the FR during a 3 week period as a training tool is associated with further improvements in walking abilities.

## Materials and Methods

The 32-year-old female patient had suffered a basilar thrombosis in 2015, resulting in a large brainstem infarction. Clinically, she presented with a spastic tetraparesis, predominantly on the right side. Sensory function tested by sensation of touch at the lower extremities was normal. She needed help for all activities of daily living and could only walk by using a high walker and support from two therapists. Her Barthel index (5) had five points. The functional ambulation category indicated 0 points (6). At the beginning of her inpatient rehabilitation in February 2020, she was able to walk 10 m.

For eliciting the FR, we used the Incedo® system (Nordic NeuroStim, Denmark). The system consists of an impulse generator, electrodes, and a sensor. The electrodes are attached to the patient's sole of the foot. The sensor is placed in the patient's shoe. As soon as the sensor recognizes the initiation of a step, a signal is sent to the impulse generator, which produces an electrical stimulation through the electrode.

Evaluation: Motor functions were tested with a 10-m walk (measured in seconds), a 2-min walk (measured in meters), and a kinematic gait analysis (RehaGait system, Hasomed Comp, Germany) over a distance of 10 m.

Evaluations were performed as baseline measurements, after 3 weeks training period and after an additional follow-up period of 3 weeks. The kinematic analysis was only conducted before and after 3 weeks of FR training. At each time point, evaluations were done with and without the Incedo® system.

During the 3-week training period, the patient received nine sessions of gait training with the Incedo® device. Each session lasted 45 min. The more affected right leg was stimulated. This training was offered in addition to the conventional rehabilitation program, which included 14 conventional gait training sessions and seven sessions on a treadmill with weight support.

After the training period, the patient was asked about her impression regarding various aspects of the electrical stimulation. A visual analogous scale ranging from 0 to 10 was used to quantify the patient's answers (**Table 3**).

## Results

At baseline, the use of the Incedo® system was associated with a higher walking velocity than without the system. After 3 weeks of training, walking with the Incedo® system was almost twice as fast compared with baseline. Moreover, after 3 weeks, she also walked faster without the Incedo® device than at baseline with the Incedo®. At follow-up, the 2-min walk results remained almost unchanged, but there was a slight deterioration in the 10-m walking test (Table 1).

**Table 1:** The 10 m walking test and 2 min walking test before and after a 3-week training period and at follow-up.

	**Baseline**	**After 3 weeks**	**Follow-up**
10 m walk with Incedo (seconds)	72	35	42
10 m walk without Incedo (seconds)	92	42	66
2 min walk with Incedo (meters)	14,5	28	30
2 min walk without Incedo (meters)	11	27	28

The kinematic analysis (Table 2) indicated that the patient's stride length and gait velocity were higher, and stride duration was shorter with FR stimulation. Consequently, the number of steps for 10 m decreased with FR activation. Only the baseline FR stimulation was associated with a considerable elevation of the foot above the ground. This effect was diminished after 3 weeks of training.

**Table 2:** Kinematic gait analysis before and after a 3-week training period.

**Kinematics**	**Baseline**		**After 3 weeks**	
	**With Incedo**	**Without Incedo**	**With Incedo**	**Without Incedo**
Steps	18	23	13	16
Double stride length (meters):	0.47	0.40	0.67	0.59
Double stride duration (seconds):	3.39	4.03	2.60	2.74
Max. foot elevation (meters):	0.11	0.03	0.03	0.03
Stance phase (%):	80.4	79.7	81	77
Swing phase (%):	19.6	20.2	19	23
Gait velocity (m/s):	0.14	0.10	0.26	0.21

At the beginning of the recent inpatient rehabilitation, the patient had been able to walk 10 m before exhaustion and required a break. At the end of the rehabilitation period, she managed to walk 100 m without a break.

Her own estimation of the electrical stimulation indicated that she did not consider it pleasant but effective and recommendable to others (Table 3).

**Table 3:** The patient's perspective: questionaire answered by the patient.

**Question**	**Answer**
Was wearing the device comfortable?	10
Was the electrical stimulation pleasant?	2
Did you feel pain during the electrical stimulation?	1
Did you feel pain after the training session?	0
Do you feel this electrical stimulation as being beneficial?	5
Do you recommend this device for others?	10

*Answers were given on a visual analogous scale ranging from “0,” corresponding to “not at all,” to “10,” corresponding to “yes, full agreement”*.

We were able to compare these results with results obtained during a preceding inpatient rehabilitation period that took place in the same rehabilitation hospital (from July until August 2018). In 2018, the patient had been able to walk 10 m without a break at the beginning and 40 m at the end of the treatment period.

## Discussion

To our knowledge, this is the first report describing the effects of an FR activation in a chronic stroke patient. Two preceding studies that focused on FR activation had been performed in subacute stroke patients. One of these studies had also used the Incedo® system (4); in the other publication, successive needle pricking on plantar and dorsal parts of the foot had been applied (7).

In our patient, even during the first utilization of the Incedo® system, gait parameters improved, indicating an immediate effect of the FR activation. The stronger elevation of the foot at baseline (compared with the measurement at the end of the training period) suggests an initial exaggeration of the FR, which habituates after repeated use. Interestingly, after 3 weeks of Incedo®-based gait therapy, the patient had improved her gait function so much that she now was even better without Incedo® compared with the baseline measurement with Incedo®. This suggests a transfer of function and less dependency on the device. Another aspect underlines this hypothesis: The difference between with and without Incedo® was much smaller after the 3-week therapy than at the beginning. Nevertheless, the fact that the patient's gait function was still better with than without the Incedo® system after the 3-week therapy suggests that the patient still benefits from the FR activation. Fortunately, the results obtained 3 weeks after the termination of the Incedo®-based therapy demonstrate a maintenance of function. The patient's judgment corresponded to what could be expected: She felt the treatment to be slightly painful and not pleasant, but moderately helpful and highly recommendable.

Compared with a previous inpatient rehabilitation 2 years earlier, the most recent inpatient treatment was associated with much stronger gains of gait abilities as determined by the distance the patient was able to walk without a break. Of course, we cannot attribute this improvement exclusively to the Incedo®-based approach since the Incedo®-based therapy had been added to the conventional rehabilitation program. The larger number of therapies itself might have produced a larger degree of improvement. Such a correlation between the number of therapies and the degree of improvement has been published earlier (8, 9).

In conclusion, our case study demonstrates that a chronic stroke patient with a severe gait impairment can benefit from activation of the flexor reflex. Of course, this single case does not allow drawing any general conclusion about the efficiency of flexor reflex activations in chronic stroke patients. However, we suggest using this technique to conduct a randomized controlled study in this patient group still suffering from severe gait deficits.

## Data Availability Statement

The raw data supporting the conclusions of this article will be made available by the authors, without undue reservation.

## Ethics Statement

Ethical review and approval was not required for the study on human participants in accordance with the local legislation and institutional requirements. The patients/participants provided their written informed consent to participate in this study. Written informed consent was obtained from the individual(s) for the publication of any potentially identifiable images or data included in this article.

## Author Contributions

CS and AS performed the experiments. CS wrote the first draft of the manuscript. CS and JL designed the experimental set-up. JL wrote the final version of the manuscript. All authors contributed to the article and approved the submitted version.

## Conflict of Interest

The authors declare that the research was conducted in the absence of any commercial or financial relationships that could be construed as a potential conflict of interest.

## References

[1] Sherrington CS. Flexion-reflex of the limb, crossed extension-reflex, and reflex stepping and standing. J Physiol. (1910) 40:28–121. 10.1113/jphysiol.1910.sp001362 16993027 PMC1533734

[2] Sandrini G Serrao M Rossi P Romaniello A Cruccu G Willer JC. The lower limb flexion reflex in humans. Prog Neurobiol. (2005) 77:353–95. 10.1016/j.pneurobio.2005.11.003 16386347

[3] Andersen OK. Studies of the organization of the human nociceptive withdrawal reflex: focus on sensory convergence and stimulation site dependency. Acta Physiol. (2007) 189:1–35. 10.1111/j.1748-1716.2007.01706.x 17439638

[4] Spaich EG Svaneborg N Jørgensen HRM Andersen OK. Rehabilitation of the hemiparetic gait by nociceptive withdrawal reflex-based functional electrical therapy: a randomized, single-blinded study. J Neuroeng Rehabil. (2014) 11:1–10. 10.1186/1743-0003-11-81 24885645 PMC4026111

[5] Hsueh IP Lee MM Hsieh CL. Psychometric characteristics of the Barthel activities of daily living index in stroke patients. J Formos Med Assoc. (2001) 100:526–32. 11678002

[6] Mehrholz J Wagner K Rutte K Meiβner D Pohl M. Predictive validity and responsiveness of the functional ambulation category in hemiparetic patients after stroke. Arch Phys Med Rehabil. (2007) 88:1314–9. 10.1016/j.apmr.2007.06.764 17908575

[7] Shen CC Lei KT Jiang JF Miao D Xiong JW. Evoking the withdrawal reflex via successive needle-pricking on the plantar and dorsal aspect of the foot increases the FMA of the lower limb for poststroke patients in brunnstrom stage III: a preliminary study. Evid Based Complement Alternat Med. (2020) 31:3805628. 10.1155/2020/3805628 32934660 PMC7479456

[8] Kwakkel G van Peppen R Wagenaar RC Wood Dauphinee S Richards C Ashburn A. Effects of augmented exercise therapy time after stroke: a meta-analysis. Stroke. (2004) 35:2529–39. 10.1161/01.STR.0000143153.76460.7d 15472114

[9] Remos-Arbeitsgruppe: Dohle C Quintern J Saal S Stephan K Tholen R Wittenberg. S2e-leitlinie Rehabilitation der Mobilität nach Schlaganfall (ReMoS). Neurol Rehabil. (2015) 21:355–494.

